# Molecular Mechanisms of Retinal Degeneration and How to Avoid It

**DOI:** 10.3390/ijms24108752

**Published:** 2023-05-15

**Authors:** Tamás Kovács-Öller, Béla Völgyi

**Affiliations:** 1 Retinal Neurobiology Research Group, Szentágothai Research Centre, University of Pécs, 7624 Pécs, Hungary; volgyi01@gamma.ttk.pte.hu; 2 Institute of Biology, Faculty of Sciences, University of Pécs, 7624 Pécs, Hungary

Vision is the most important sensory modality in vertebrates in general, and as such, it is the most feared sense to lose. The retina is the site for visual perception, and we desperately need new tools and advanced therapeutic regimens to avoid retinal degeneration or at least slow down ongoing deteriorating diseases, including glaucoma, diabetic retinopathy, age-related macular degeneration, myopia, retinal vascular disease, traumatic brain injuries, and many others. In our Special Issue, entitled “Molecular Mechanisms of Retinal Degeneration and How to Avoid It”, we show recent advances in the field of retinal neuroscience, with an emphasis on diseases.

Following a primary insult, including retinal degenerative (RD) diseases, most retinal cell types are involved in the progression of tissue deterioration and thus suffer from the corresponding side effects. Thus, both retinal neurons (ganglion cells, amacrine cells, bipolar cells, horizontal cells, and photoreceptors) and non-neuronal cells (astrocytes, microglia, and Müller cells) are continuously dying and thereby contributing to the progression of vision loss. Comprehending the molecular mechanisms by which retinal diseases alter their function is crucial. Our Special Issue aims to broaden the knowledge of disease phenotypes at all investigation levels since there is still a paucity of information in identifying potential targets for regeneration, potentially restoring vision, or at least promoting cell survival. Therefore, this collection of articles covers various aspects of the field.

The article by **Lin and colleagues** [1] evaluates and confirms the effects of ***myopia*** on eye growth, taking three basic elements of the neurovascular unit into account: blood vessels, astrocytes, and ganglion cells. In this fascinating study, the authors used the marmoset monkey (*Callithrix Jacchus*) as an animal model, whose eyes share many morphological and functional features with those of the human eye. Given that myopia is one of the most widespread human eye diseases, the use of monkeys for this study provides great clinical relevance; ~30% of the human population is already affected by myopia, and the incidence rate is still growing, with a grim perspective predicting that over half of the human population will suffer from it in the coming decades [2]. Lin and colleagues here find that retinas with lens-induced myopia showed an aberrantly thin neurofilament layer. This change was accompanied by a reduced number of astrocytes, which was associated with a lowered peripheral branching of the retinal vasculature.

***Retinitis pigmentosa*** is a major blinding disease affecting photoreceptors. Among the 90 genes associated with RP, “progressive rod-cone degeneration” (PRCD) is one of those affecting both dogs and humans. The genetic defect induces the expression of a short protein altered by amino acid exchange (R17C). **Myers and colleagues** [3] showed how this alteration plays a role in regulating the packaging of rhodopsin into outer segment disc membranes. The R17C mutation takes place in the PRCD protein at the polybasic region (PBR), composed of three arginine residues following the transmembrane helix. In addition, the authors also showed that the palmitoylation of cysteine in the second amino acid position is essential for the proper localization of the PRCD molecule due to its membrane-anchoring role in the outer segment of photoreceptors. The acylation of the same site might also contribute to the stability and trafficking of the PRCD protein, which can also lead to the RD phenotype.

Cell cultures are always essential tools to study diseases. Therefore, creating new in vitro models for the retina is crucial for understanding the ***environmental effects*** on cells outside the protection of the bony skull. **Gaddini and colleagues** [4] investigated how γ-ray exposure from various sources can have detrimental effects on retinal cells, including glial cells such as astrocytes and microglia. In their model system, they demonstrated a potential protective effect of low-dose pre-irradiation, resulting in reduced cellular and DNA damage.

***Age-related macular degeneration*** (AMD) is one of the significant retinal diseases affecting the central part of the retina responsible for sharp, detailed vision. AMD is a progressive disease that can lead to vision loss in people over the age of 50, thus gaining more and more relevance in aging societies. **Dörschmann** and the Klettner group [5,6] provided new evidence on how a group of plant polysaccharides, fucoidans, are capable of alleviating the effects of AMD. In their elegant back-to-back studies, they analyzed the true potential of fucoidans in retinal medicine. In the first study, they showed that Fuc1 from brown algae, Laminaria hyperborea, induced antiangiogenic and anti-inflammatory activities while retaining the impaired RPE cellular functions proven by a gene expression platform. In the second article, two Saccharina latissima fucoidans were tested for their AMD-related biological effects in primary porcine retinal pigment epithelium (RPE), human RPE cell line ARPE-19, and human uveal melanoma cell lines (OMM-1). Both compounds reduced angiogenic cytokines and showed anti-inflammatory and complement-inhibiting properties; thus, potential effects on gene expression and RPE functions need to be considered for further research.

In an additional effort to offer new modalities to treat AMD, **Wang and Urrutia-Cabrera et al.** [7] also developed a new model to study AMD as well as other retinal diseases affecting the RPE. Their RPE model integrated CRISPR interference for gene repression by generating a stable ARPE-19 cell line expressing dCas9-KRAB, which utilizes an inactive SpCas9 (dCas9) coupled with the transcriptional repressor domain Kruppel-associated box (KRAB). They tested it with specific sgRNAs for the knockdown of transmembrane protein 97 (TMEM97), reducing reactive oxygen species (ROS) levels and exerting protective effects.

As part of the central nervous system, the retinal tissue responds to changes in the environment, and upon destructive insults, it responds with the initiation of degenerative processes. The retina is also prone to death and/or survival factors released by other brain areas affected by the primary insult. In a compelling research line, **Kovacs-Öller and colleagues [8]** investigated the differential effects of severe and random mild ***traumatic brain injuries*** (sTBI, rmTBI) in the retina. They showed that even mild injuries can lead to microglial activation in the ganglion cell layer on a level reminiscent of those following sTBI. In addition to microglial activation, the increased level of Caspase3 expression indicated a widespread effect of rmTBI in many cell types with astrocytes and microglial cells among those reacting first.

In addition to direct environmental effects, many degenerative retinal diseases can develop into critical visual impairment or even vision loss. This Special Issue highlighted many of these factors and also pointed out some of the research avenues that can potentially lead to alternative treatment modalities or even to cures for such degenerative processes. Ophthalmologists desperately need new methods and drugs to better understand vision and to treat visual diseases; therefore, the importance of the work presented here, along with other research lines focusing on the progression of degenerative changes in the retinal tissue, cannot be overemphasized. The future of many visually impaired, not to mention other diseases somehow linked to the retina, potentially lies in the hands of, among many other scientists, the authors who contributed to this Special Issue. Such work can be used for translational research and, eventually, in clinical practice.
